# Assessment of Knowledge and Practice towards Hepatitis B among Medical and Health Science Students in Haramaya University, Ethiopia

**DOI:** 10.1371/journal.pone.0079642

**Published:** 2013-11-21

**Authors:** Yonatan Moges Mesfin, Kelemu Tilahun Kibret

**Affiliations:** 1 Department of Public Health, College of Medical and Health Science, Haramaya University, Harar, Ethiopia; 2 Departments of Public Health, College of Medical and Health Science, Wollega University, Nekemte, Ethiopia; Kliniken der Stadt Köln gGmbH, Germany

## Abstract

**Introduction:**

Hepatitis B (HB) is a serious infection that affects liver and caused by hepatitis B virus (HBV). HB is a serious global public health problem and the health professionals are most at risk. It is contagious and easy to be transmitted from one infected individual to another by blood to blood contact, mother to child, unprotected sexual intercourse, sharing of eating utensils and other barber shop and beauty salon equipment. The aim of this study was to assess knowledge and practices about transmissions and prevention of hepatitis B among medical and health science students on clinical attachment in Haramaya University.

**Methods and Findings:**

A cross sectional study was conducted among 322 health science and medical students who are starting clinical attachment (year II, III, IV, V and IV) from February 1–15, 2013. Self-administered structured questionnaire was used to collect information. Out of 322 distributed questionnaires, 322 were returned with a response rate of 100.0%. Majority of the students (91%) were in the age group 20–24 and 232 (72%) of the respondents were male. Majorities (95.3%) of students were not fully vaccinated against Hepatitis B and 48.4% of the students were not aware about the availability of post exposure prophylaxis for HB. Mean scores for knowledge and practice were 11.52±2.37 and 2.76±1.1 respectively. Significant and positive linear correlations between knowledge-practice (r = 0.173, p = 0.002) was observed. Study department was significantly associated with mean knowledge and practice of study respondents.

**Conclusion:**

This study indicates that lack of awareness about Hepatitis B, its route of transmission and modes of prevention among the medical students entering into the profession. Similarly, 95.3% the students were not fully vaccinated against Hepatitis B, which makes them vulnerable to the disease.

## Introduction

**Hepatitis B (HB)** is a serious blood born infection that affects the liver and caused by hepatitis B virus (HBV). It is infectious and the most common cause of chronic hepatitis, liver cirrhosis and hepato-cellular carcinoma [1]. Hepatitis B is a very important public health problem affecting almost 10% of the world population [2]. According to 2009 WHO report, about 2 billon people are affected with HB worldwide, more than 350 million suffered from chronic lifelong infection and, more than one million of individuals die because of cirrhosis and liver cancer every year [3].

HBV is contagious and easy to be transmitted from one infected individual to another by blood to blood contact, mother to child, unprotected sexual intercourse, sharing of eating utensils and other barber shop and beauty salon equipment [4]. The main transmission routes include prenatal infection, skin and mucous membrane infections caused by contaminated blood or body, sexual contacts and injection drug abuser. In addition, tattooing, ear piercing, acupuncture, dialysis, and even using a syringe can be the source of infection. In volunteer blood donors, the prevalence of anti HB reflection ranges from 5–10%. But the prevalence is higher in lower socio-economic status, older age group and those persons exposed to blood products [5], [9]. Prevalence of infection, modes of transmission and human behavior conspire to geographically different epidemiologic patterns of HB infection [6].

The practice of modern medicine have contributed a lot in the increase of the case and spreading of blood born diseases like Human immune deficiency virus and HBV due to lapse in the sterilization technique of instruments and improper hospital waste management as 10 to 20% health care waste is regarded hazardous [7].

Prevention against any disease is proportional to knowledge, attitude and practice (KAP) of the population and reflection of the importance that is paid to health related issue by the society. Health care workers should familiarize themselves with “universal precautions”, which is defined by Center for Disease Control, as a set of precautions designed to prevent transmission of Human immunodeficiency virus (HIV), HBV, and other blood-borne pathogens when providing first aid or health care. Under universal precautions, blood and certain body fluids of all patients are considered potentially infectious for HIV, HBV and other blood borne pathogens [8].

A safe and effective vaccine against HBV is available since 20 years and is effective in preventing infection and the serious consequence of hepatitis including liver cancer and cirrhosis when given before or after exposure [9]. In Ethiopia, the Expanded program for immunization program (EPI) delivers eight vaccines to protect children against the following serious childhood illnesses: tuberculosis, poliomyelitis, diphtheria, pertussis, tetanus, *Haemophilus influenza-B* (Hib) infections, hepatitis B disease and measles. The hepatitis B vaccines are new vaccines introduced into the EPI programme in Ethiopia in 2007. Since 1990 new HB infections among children and adolescents have dropped by more than 95% and by 75% in other age group. Vaccination gives long term protection from HB infection, possibly life-long. KAP surveys are representative of a specific population to collect information on what is known, believed and done about a particular topic, and are the most often used study tool in health-seeking behavior research [10]. Knowledge is usually assessed to see how far community knowledge corresponds to biomedical concepts [11]. Practices in KAP surveys usually inquire about preventive measures or different health care options. Normally, hypothetical questions are asked, so it permits statements about actual practices, rather, it yields information on people’s behaviors or on what they know should be done [12].

Medical and health science students, being part of the health care delivery system, are exposed to the same size of risk as other health care workers when they come in contact with patients and contaminated instruments. They are the first level of contact between patients and medical care. They are expected to undertake activities related to patient care with the beginning of their clinical years. As on date, very few studies have been conducted to find out the knowledge and practice of medical students about Hepatitis B in Ethiopia. This study assessed the knowledge and practice of students towards HB transmission and prevention among medical and health students in Haramaya University.

## Materials and Methods

### Study Area and Period

This cross- sectional study was conducted from February 1–15, 2013 in College of medical and health science, Haramaya University, largest and oldest educational institution in the country. The University is running 50 degree, 50 masters and 13 PhD programme. The University has 12 colleges (including the health science and medical collage that has 8 departments). It has one teaching referral hospital, one health center and one clinic. The college of medical and health science is located in Harar town 25 kilometer away from the main campus and 500 kilometer away from Addis Ababa.

All Medical and Health Science students of Haramaya University who were on clinical attachment included in this study. The students who were not on clinical attachment, those who have mental illness, seriously ill and blind were excluded from the study.

### Sample Size Determination

The sample size was calculated by using Epi Info 3.5 software package based on single population proportion formula (estimated prevalence rate of 50% konwledge, margin of error 5% and a 95% confidence interval). The calculated sample size was 384. Since the source population is less than 10000, we used correction formula and adding 10% for none response, the resulting minimum sample size was 322.

### Sampling Method

Study participants were selected using systematic sampling technique. First we stratified students based on year of study and department. Second, the sample size was distributed proportionally to each year of study based on the student population they have. Finally using students list respondents were selected by systematic random sampling. In case of absenteeism the next number was included in the study.

### Variables

Knowledge and practice of students towards transmission and prevention of Hepatitis B virus (HBV) were considered as dependent variables and the independent variables were age, sex, and residential area, and marital status, year of study and departments of the study population.

### Data Collection Process

A self-administered structured questionnaire was used to collect information about the socio-demographic characteristics of respondents, knowledge towards transmission and prevention method of hepatitis B virus and practice towards prevention HBV. The English version of the questionnaire was used to collect the information from the respondents. The questionnaire was pretested on 5% of pre-clinical students in the college other than the study participants and necessary changes were implemented. Training was given for data collectors and the overall data collection activities were supervised by principal investigator. The participants were anonymously responded to the items on the questionnaire.

### Data Analysis

Data was checked for completeness and consistency. Coded data were entered and cleaned using Epi Data software and analyzed using SPSS version 20.0 (SPSS, Chicago, IL, USA). Descriptive statistics were conducted using frequencies and proportions. Bivariate and multivariate analyses were carried out using logistic regression to examine the relationship between the outcome variable (mean knowledge and practice) and selected socio-demographic factors. Adjusted and unadjusted odds ratios (OR) and their 95% confidence intervals (CIs) were used as indicators of the strength of association. A p-value of 0.05 or less was used as cut off level for statistical significance.

### Ethical Consideration

The research protocol was approved by the medical and Health Sciences College Research Ethics review Committee of the Haramaya University. Then informed written consent was obtained from each study participants. Individual participant consent was waived/by the Ethics Committee. That means the committee save grads not a rule to be obeyed. Written informed consent was obtained from the immediate caretaker, or next of kin, prior to inclusion, on behalf of children participating in the study. Moreover confidentiality assured for all the information provided and personal identifiers were not included on questionnaire.

## Results

A total of 322 students belonging to 2^nd^, 3^rd^, 4^th^, 5^th^, and 6^th^ year from six different departments were approached for the study and all of them were participated in the study making response rate of 100%. Majority of the students (91%) were from the age group 20–24 and 232 (72%) of the respondents were male. Majority, 307 (95.3%) of the study participants were single in marital status and more than half; (55.6%) of the respondents came from urban area **(** **Table 1** **)**.

**Table 1:** Characteristics of the study participants, Haramaya University, 2013.

Characteristics	Frequency	Percent (%)
**Age**		
**15–19**	8	2.5
**20–24**	293	91
**25–29**	21	6.5
**Sex**		
**Male**	232	72
**Female**	90	28
**Department**		
**Medicine**	82	25.5
**Public health officer**	91	28.3
**Nurse**	72	22.4
**Midwifery**	40	12.4
**Psychiatry**	25	7.8
**Medical laboratory**	12	3.7
**Year of study**		
**Second year**	39	12.1
**Third year**	86	26.7
**Fourth year**	163	50.6
**Fifth year**	18	5.6
**Sixth year**	16	5.0
**Marital status**		
**Single**	307	95.3
**Married**	10	3.1
**Divorced**	5	1.6
**Residence**		
**Urban**	179	55.6
**Semi-urban**	73	22.7
**Rural**	70	21.7

### Knowledge towards Hepatitis B Transmission and Prevention

Knowledge was assessed by questions focusing on sign and symptoms, transmission, treatment and prevention. Each response was scored as ‘yes’ or ‘no’. The scoring range of the questionnaire was 16 (largest) to 0 (smallest). A cut off level of <12 was considered as poor whereas > = 12 was considered as adequate knowledge about HB. Knowledge scores for individuals were calculated and summed up to give the total knowledge score. Out of the 322 participants, 141 (43.8%) were within the poor knowledge range whereas 181 (56.2%) showed adequate knowledge about HB. Poor knowledge was apparent in responses to questions relating to transmission (question 7–10). Nearly half; 156 (48.4%) of the study participants were not know that HB has post exposure prophylaxis and the mean knowledge score for the entire study cohort was 11.52±2.37 **(** **Table 2** **)**.

**Table 2:** Responses of the study participants to Hepatitis B knowledge items Haramaya university, 2013 (N = 322).

No	Hepatitis B Knowledge Items	Yes	No
		N (%)	N (%)
**1**	Have been thought about HBV and its vaccine	261 (81.1)	61 (18.9)
**2**	Can HBV affect any age group	287 (89.1)	35 (10.9)
**3**	Do you think that HBV can affect liver?	304 (94.4)	18 (5.6)
**4**	Do you think that HBV can affect other organ other than liver?	210 (65.2)	112 (34.8)
**5**	Is jaundice one of the common symptom of HBV?	274 (85.1)	48 (14.9)
**6**	Can Hepatitis B be transmitted by contaminated blood and blood products?	289 (89.8)	33 (10.2)
**7**	Can Hepatitis B be transmitted by un-sterilized syringes, needles and surgical instruments?	231 (71.7)	91 (28.3)
**8**	Can Hepatitis B be transmitted by unsafe sex?	211 (65.5)	111 (34.5)
**9**	Can Hepatitis B be transmitted from mother to child?	180 (55.9)	142 (44.1)
**10**	Can Hepatitis B be transmitted by contaminated water/food prepared by personsuffering with these infections?	71 (22)	251 (78)
**11**	Can hepatitis B transmitted through skin contact	155 (48.1)	167 (51.9)
**12**	Do you think HBV has laboratory test?	304 (94.4)	18 (5.6)
**13**	Is Hepatitis B curable/treatable?	152 (47.2)	170 (52.8)
**14**	Could we prevent HB transmission?	314 (97.5)	8 (2.5)
**15**	Is vaccination available for Hepatitis B?	300 (93.2)	22 (6.8)
**16**	Do you think that HBV has post exposure prophylaxis?	166 (51.6)	156 (48.4)

Note: Knowledge was assessed by giving 1 to correct answer and 0 to the wrong answer. The scale measured knowledge from maximum 16 to minimum 0. Scores <12 were taken as poor, ≥12 as adequate knowledge of Hepatitis B. Mean knowledge was 11.52±2.37.

### Assessment of Practices towards Hepatitis B Prevention

Practices towards HB prevention were assessed by asking five questions as shown in Table 3. Each question was labeled with good or poor practice. A score of 1 was given to good while 0 was given to bad practice with a score range of largest of 5 to a smallest of 0. The scale classified practice as good with score >3 and poor ≤3. Majority of the respondents, 276 (85.7%) never screened for HB and 279 (86.6%) stated a negative immunized status against HB. It was interesting to know that nearly one third 102 (31.7%) of the respondents never asked for screening of blood and blood products before transfusion, and 53 (16.5%) of the respondents never asked for a new syringe when required. Majority, 245 (76.1%) of the respondents were never participated in any education program on HB. The mean score for HB related practices was 2.04±1.15 revealing poor practices among the study participants. Out of 322 participants, 43 (13.4%) were vaccinated against HBV. In the vaccinated group, 15 (4.7%) completed all 3 doses of their vaccination schedule and remaining 28 (8.7%) students were incompletely vaccinated. Reasons for not getting vaccinated were lack of information in 67 (20.8%) students, no need was felt by 9 (2.8%) students, 15 (4.7%) had fear of injection and 45 (14%) said they ignorance **(** **Table 3** **)**.

**Table 3:** Practice related to Hepatitis B prevention among health science and medical students of Haramaya university, 2013 (N = 322).

Hepatitis B practice item	Yes	No
	N (%)	N (%)
**Have you done screening for Hepatitis B?**	46 (14.3)	276 (85.7)
**Have you got yourself vaccinated against Hepatitis B?**	43 (13.4)	279 (86.6)
**Do you ask for screening of blood before transfusion?**	220 (68.3)	102 (31.7)
**Do you ask for a new syringe before use?**	269 (83.5)	53 (16.5)
**Have you ever participated in health education program related to Hepatitis B?**	77 (23.9)	244 (75.8)

Note: Practice was assessed by giving 1 to positive and 0 to negative practice. The scale classified practice as good with score >3 and poor ≤3. Over all the respondents reported to have poor practice towards Hepatitis B with mean score of 2.76±1.1.

### Association of Demographic Characteristics and Mean Knowledge and Practice Scores

The association of demographic characteristics and mean Knowledge and practice scores using multivariate logistic regression is presented in Table 4. Among the demographic variables, study department and sex of the respondents were significantly associated with both mean knowledge and practice scores. The difference of mean knowledge and mean practice score across study department of the students were found to be significant at 95% confidence interval (P = 0.007) and (P = 0.001). Being psychiatry and medical laboratory students is statistically associated with poor knowledge towards transmission and prevention of HB and poor practice towards prevention of HB as compared to medical students. Even though year of study is not statistically associated with poor mean knowledge score towards transmission and prevention of HB (P = 0.17), being second year students or starting clinical attachment in early year of study (2^nd^) is positively and significantly associated with poor mean knowledge of HB (OR, 11.7 and P = 0.042).

**Table 4:** Comparisons of Demographic Characteristics and Mean Knowledge and Practice Score of participants, Haramaya University, 2013.

Description	N (322)	Knowledge score	P-value	Practice score	P-value
		Mean ± SD		Mean ± SD	
**Age**			0**.09**		**0.805**
**15–19**	8	10.38±4.31	1.00	1.38±0.74	1.00
**20–24**	292	11.52±2.28	0.961	2.04±1.15	0.999
**25–29**	21	12.19±2.29	0.078	2.43±1.23	0.999
**Sex**					
**Female**	89	11.06±2.83	**0.025**	1.90±1.07	**0.087**
**Male**	232	11.72±2.13		2.10±1.17	
**Study Department**			**0.007**		**0.001**
**Medicine**	82	12.53±1.92	1.00	2.00±83	1.00
**Public health officer**	91	12.03±1.87	0.878	1.80±1.01	0.443
**Nurse**	71	10.91±2.78	0.527	1.97±1.27	0.255
**Midwifery**	40	11.55±2.14	0.552	3.02±1.31	0.006
**Psychiatry**	25	9.44±1.52	0.001	1.76±1.16	0.716
**Medical laboratory**	12	8.91±2.35	0.049	2.00±1.04	0.726
**Year of study**			**0.17**		**0.338**
**6^th^ year**	38	13.06±1.73	1.00	2.06±0.77	1.00
**5^th^ year**	86	12.22±2.23	0.162	2.12±0.60	0.430
**4^th^ year**	163	12.01±2.04	0.164	2.08±1.07	0.871
**3^rd^ year**	18	11.01±1.90	0.073	1.88±1.25	0.977
**2^nd^ year**	16	9.73±3.43	0.042	2.24±1.49	0.998
**Marital status**			**0.223**		**0.158**
**Single**	306	11.49±2.37	1.00	2.04±1.15	1.00
**Married**	10	12.30±1.76	0.267	2.40±1.07	0.139
**Divorced**	5	12.80±1.92	0.162	1.80±1.30	0.181
**Residence**			**0.351**		**0.222**
**Urban**	178	11.72±2.38	1.00	2.12±1.11	1.00
**Semi urban**	73	11.34±2.34	0.461	1.95±1.05	0.144
**Rural**	71	11.25±2.29	0.152	1.96±1.32	0.668

Multivariate logistic regression showed that study department significantly associated with poor practice towards prevention of HB (P = **0.001)**. There is no variation towards poor practice in prevention of HB across year of study **(** **Table 4** **)**.

### Correlation between Mean Knowledge and Practice of the Students towards HB

Correlations were interpreted using the following criteria: 0–0.25 = weak correlation, 0.25–0.5 = fair correlation,0.5–0.75 = good correlation and greater than 0.75 = excellent correlation [13]. This study showed significant positive but weak linear correlations between knowledge and practice (r = 0.173, p = 0.002). The result reaffirms the relationship between knowledge and practice with infection control measures even though the correlation is weak in this study.

The positive correlations between knowledge-practice in this study reaffirm the relationship between knowledge and practice with infection control measures. It is concluded that adequate knowledge can lead in good practices.

## Discussion

The current study sought to evaluate knowledge and practice towards HB among medical and health science students who started clinical attachment. Results of the study showed poor knowledge and practice towards HB. The mean knowledge score was *11.52±2.37* indicating low level of knowledge towards HB among the study cohort.

Scientific knowledge about HBV transmission is essential for medical students. They can take proper protection during their clinical posting as HBV is 50 times easier to transmit than HIV [14]. The knowledge about transmission of Hepatitis B through sexual route (65.5%) by used needles and syringes (71.7%) by blood transfusion (89.8%) was high. But through vertical transmission (55.9%) and contaminated water/food prepared by person suffering with these infections (22%) was low among overall medical and health science students. Among study participants, 62.4% of them know that HB can cause liver cancer and affect other organs other than liver, again a major sign of concern. These results are in line with the findings from studies reported from B.J.Medical College, Ahmadabad, and Gujarat, India; where majority of the medical students had correct knowledge on mode of transmission [15].

In the present study, 13.4% of the students received one or more doses of hepatitis B vaccine and among which 4.7% of the students were fully vaccinated against Hepatitis B.

This was lower than the vaccination status of 87.8% study done at Muhammad Medical College Mirpurkhas [16], 29.3% reported among medical students of B.J. Medical College [15], 35% reported in civil hospital of 60 laboratory technicians [17], 88.1% in **study done at two national/regional congresses and two university hospitals in Iran (6)** [**18**] **,** 63% reported from India among medical students and 42% reported among medical students of Lahore [19]. The most frequent reason for not getting vaccinated in the present study was inaccessibility 166 (51.6%), lack of motivation 67 (20.8%) and followed by ignorance 45 (14%) or fear of injection 15 (4.7%). The finding is consistent with a study result from medical college of Mirpurkhas, Pakistan [16]. These are serious issues and baseless reasons and need to be improved by education. This study also showed that 89.4% of the respondents have poor practice towards prevention of HB.

The positive correlations between knowledge and practice in this study re affirm the relationship between knowledge and practice with infection control measures. It is concluded that adequate knowledge can lead to good practices. The findings are in line with the results presented by the study in India [20] and Pakistan [21].

## Conclusion and Recommendation

The present study concludes that there is poor knowledge among the medical and health science students entering into the profession practice about the hazards of Hepatitis B, its mode of transmission and prevention. Moreover, majority (95.3%) the students were not fully vaccinated against Hepatitis B and 48.4% of the students were no aware about the availability of post exposure prophylaxis for HB, which made them more vulnerable to the disease in their professional life and 75.8% of the students were not participated in health education program related to Hepatitis-B. Study department is the strong independent correlates for poor knowledge of the students towards transmission, prevention of HB and practice in preventing HB.

Since medical and health science students are at increased risk of acquiring needle stick injury, and exposed to blood and blood products in their professional practice, medical and health science students should be vaccinated upon entry into the medical college. Medical and Health Science Colleges should have Occupational or Student Health Departments that must take responsibility for HBV testing, vaccination, monitoring vaccine response and providing post-exposure prophylaxis. It is also recommended that a policy be implemented for complete vaccination and giving training on infection prevention for all medical and health science students before they start clinical attachment (professional practice) in all medical and health science colleges in the country despite of study department (profession). It’s also advisable to make sure vaccine availability and accessibility.
